# Accountability and equity in primary health care: co-creating user-centered evaluation systems for public-private partnerships

**DOI:** 10.3389/fpubh.2026.1938730

**Published:** 2026-09-29

**Authors:** Vera Schattan P. Coelho, Frederico Haddad

**Affiliations:** 1 Citizenship, Health and Development Group, Brazilian Center of Analysis and Planning (CEBRAP), Federal University of ABC, Santo André, Brazil; 2 Centro Brasileiro de Analise e Planejamento, São Paulo, Brazil; 3 Citizenship, Health and Development Group, Brazilian Center of Analysis and Planning (CEBRAP), Federal University of Maranhão, São Luís, Brazil

**Keywords:** plural health systems, health equity, accountability, primary health care, user-centered evaluation, SUS Brazilian national health system, public-private partnerships, patient and public involvement (PPI)

## Abstract

**Background:**

Brazil’s Unified Health System (SUS) is one of the world’s largest universal health systems and operates through a plural governance arrangement involving public, philanthropic, and community actors under public coordination. In Primary Health Care (PHC), non-profit Social Health Organizations (OSS) are increasingly responsible for managing public healthcare facilities in large urban municipalities. However, evaluation systems used by public managers remain largely focused on service delivery indicators, with limited consideration of equity and user experience.

**Aim:**

To examine how participatory approaches can broaden the informational foundations of evaluation, strengthen social accountability in contractual governance, and support more equity-oriented primary health care.

**Methods:**

We conducted a comparative cross-sectional qualitative study embedded in a multi-stage participatory research process in two large urban Brazilian municipalities. Data collection included a review of policy documents and literature, semi-structured interviews with public officials, OSS managers, health professionals, health council representatives, and focus group discussions with users and health council representatives. Findings from these stages informed an iterative co-creation process to develop a user-centered questionnaire.

**Results:**

Documentary analysis and key informant interviews showed that evaluation systems in both municipalities had developed substantial capacities for monitoring service production, coverage, operational performance and contractual compliance, while user experience, responsiveness and equity remained less systematically incorporated. Drawing on these materials, we developed an equity framework organized around three broad evaluative dimensions—Responsiveness to Population Needs and Territorial Conditions, Sociocultural Dimensions of Care, and Comprehensive Care for the Person. Focus group discussions further showed how these gaps were experienced in practice, particularly through barriers related to access, communication, continuity, respect and responsiveness to diverse needs. Finally, findings from the documentary analysis, interviews and focus group discussions were integrated with the equity framework and translated into a proposed user-centered questionnaire.

**Conclusion:**

Co-creation broadens the informational foundations of evaluation by translating users’ experiential knowledge into actionable measures for performance assessment and quality improvement. This has the potential to strengthen accountability by incorporating forms of knowledge that are typically absent from conventional performance frameworks.

## 1 Introduction

Health systems increasingly operate through governance arrangements in which public authorities finance, regulate and coordinate services delivered by a plurality of public, private, philanthropic and community actors (1). In Primary Health Care (PHC), this plurality has often been justified as a strategy to expand access, increase managerial flexibility and address limitations in direct public provision. Contracting with non-state providers has become a central instrument in these arrangements, enabling governments to set performance requirements and establish accountability mechanisms (2–4).

Brazil’s Unified Health System (Sistema Único de Saúde—SUS) provides an important setting for examining these dynamics. As a universal, tax-funded system grounded in the right to health, the SUS combines national policy frameworks with decentralized implementation at the state and municipal levels, depending on the type of care (5). PHC, largely managed at the municipal level, has played a central role in expanding access, particularly through the Family Health Strategy (6, 7). However, municipalities have adopted different governance arrangements. In large urban centers, non-profit Social Health Organizations (Organizações Sociais de Saúde—OSSs) have assumed an increasingly important role in the delivery and management of public PHC services, bringing questions of performance, accountability and evaluation to the forefront (8, 9).

Contracting generates demands for performance measurement, reporting, legal oversight and managerial coordination, which in turn shape what becomes visible and counts as relevant performance. Evaluation systems developed in contractual settings often privilege dimensions that are easier to define, standardize, compare and audit, such as service production, coverage, team composition, operational compliance and selected clinical indicators. While important for management and accountability, these measures only partially capture PHC objectives.

A central challenge is that dimensions related to quality of care, user experience, responsiveness and equity remain insufficiently incorporated into routine evaluation systems. This reflects the difficulty of operationalizing aspects of care that are relational, territorial and experiential in nature and are not easily captured through administrative data or production indicators. Equity requires attention to how social, racial, gender, territorial and economic inequalities shape access, continuity, communication and users’ capacity to benefit from services (10, 11). These dimensions are central to PHC, where care depends on sustained relationships between users, professionals, community and local contexts.

Although the SUS has a strong institutional tradition of social participation, through health councils and conferences, there are persistent gaps between formal participation and its influence on decision-making, particularly in areas such as contracting and performance evaluation (12–15). As a result, social accountability mechanisms may generate relevant information about users’ experiences, service needs and territorial problems without this information being systematically translated into evaluation frameworks or contractual governance processes.

One possible way of addressing this gap is to move beyond consultative forms of participation and incorporate users’ perspectives directly into evaluation processes (16). Participatory research methodologies have long sought to involve affected communities in the production of knowledge, the definition of priorities and the development of solutions grounded in lived experience (17, 18). More recently, Patient and Public Involvement (PPI), co-design and co-creation approaches have gained prominence within health systems as strategies for incorporating users’ perspectives into the design, delivery and evaluation of services (19, 20). In PHC, these approaches are particularly relevant because services are closely embedded in users’ everyday lives and depend heavily on communication, trust, continuity, and contextual adaptation.

Building on this discussion, the initial research set out to explore ways of strengthening the accountability of contractual arrangements to service users. Through this process, it became clear that broadening the informational foundations of evaluation - by integrating users’ perspectives into the definition of evaluative criteria - was a promising pathway forward. These insights informed a collaboratively developed Action Plan that translated the findings of the policy analysis into priorities for subsequent research activities aimed at advancing this pathway. The Action Plan guided several subsequent participatory research stages through which gaps in existing evaluation systems were further explored, ultimately informing the co-creation of a questionnaire.

Developed within the SUS, the instrument seeks to capture features of care quality, equity and local responsiveness that are relevant from users’ perspectives but remain underrepresented in existing evaluation systems. It is designed to serve as a tool for potential routine use by public officials, OSS managers and health councilors involved in contractual governance. In doing so, the study explores how closer integration between participation and evaluation can strengthen social accountability and support more equity-oriented primary health care. The next section describes the study design and methods.

## 2 Methods

### 2.1 Study design

We conducted a comparative cross-sectional qualitative study to examine and strengthen equity-sensitive evaluation in PHC services delivered through contractual arrangements with non-state providers. The study was embedded in a multi-stage participatory research process – combining normative and documentary analysis, qualitative interviews, focus group discussions and iterative co-creation workshops – through which stakeholders contributed to identifying evaluation priorities, interpreting findings and collaboratively developing the evaluation framework and questionnaire. Although data were collected sequentially across the different stages of the study, this reflects the iterative nature of the research process rather than repeated measurement of the same respondents over time. Figure 1 presents the research process.

Box 1 Local contracting landscape in Brazil’s Unified Health System (SUS).

**Table:** 

SUS and PHC governance	Brazil’s Unified Health System (Sistema Único de Saúde – SUS) is a tax-funded universal health system. National authorities establish broad policy and financing frameworks, while municipalities play a central role in organizing and delivering primary health care.
Social Health Organizations (OSSs)	OSSs are private non-profit organizations contracted by public authorities to manage publicly funded health services. Facilities remain part of the SUS and services remain publicly funded and free at the point of use; contracting changes the management and delivery arrangement rather than the public character of the service.
Contracting PHC services in São Paulo	Contracting expanded progressively from the early 2000s and OSSs now manage more than 84.5% of the PHC services (21). Contracts are territorially organized, with providers responsible for public PHC facilities within defined health regions, and are accompanied by structured targets, monitoring and performance-assessment mechanisms.
Contracting PHC services in Fortaleza	Direct public provision remains predominant, while OSSs manage approximately 30% of PHC units. In parallel, a system-wide contractual arrangement covers care-related macro-processes - including information systems, scheduling, pharmacy logistics and medical records - across virtually the entire municipal PHC network (9).
Accountability Arrangements	Contracted providers operate within public managerial, legal and social accountability arrangements involving municipal health authorities, oversight institutions and participatory bodies such as health councils.

**Figure 1:**
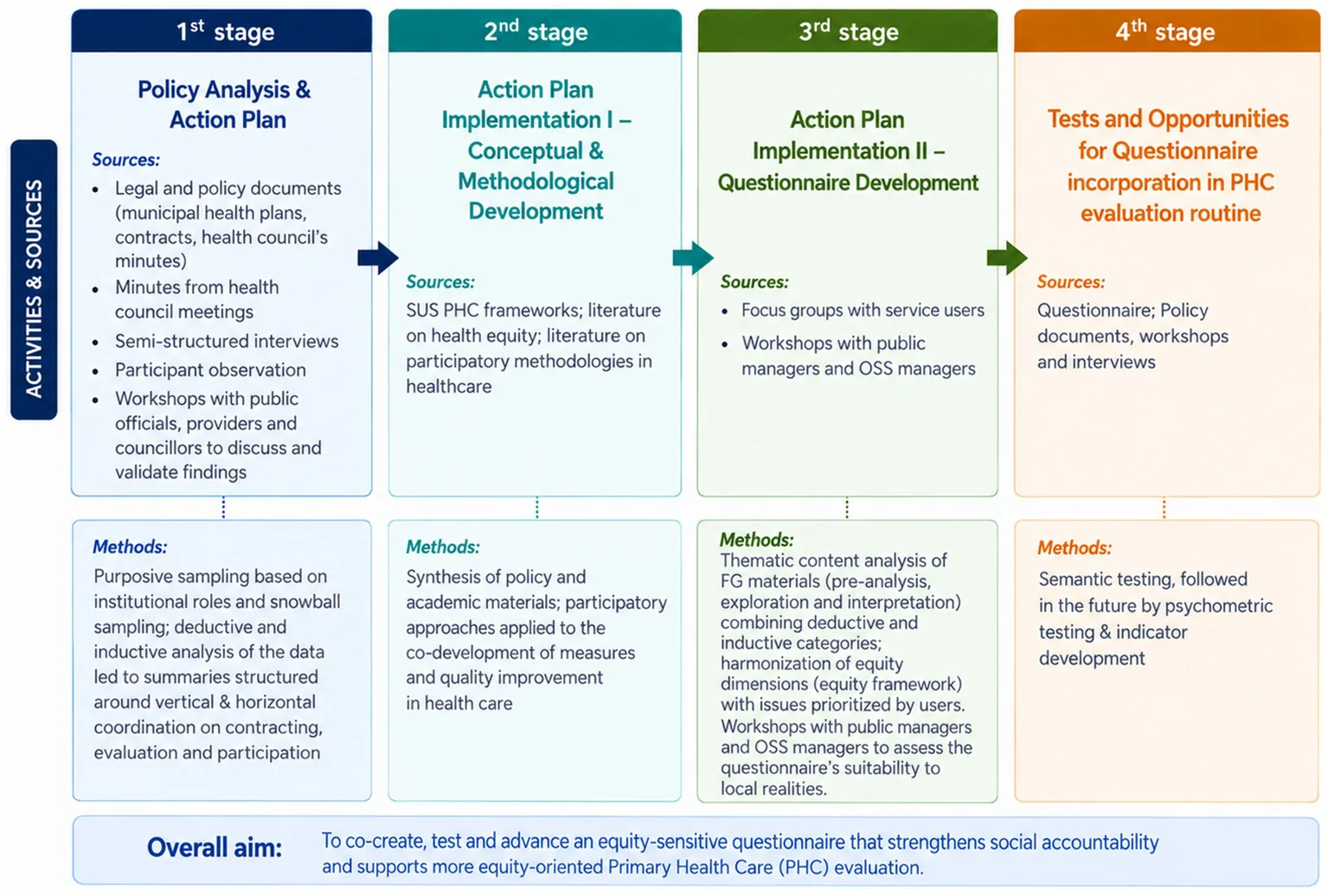
Research process. Source: Prepared by the authors, NCSD, 2026.

The study is part of the Community Voices in Health Governance (COMPLUS) project, an international initiative examining how community participation can strengthen learning, accountability and service improvement in plural health systems.

### 2.2 Settings

The study was conducted in São Paulo and Fortaleza, two of Brazil’s largest municipalities and major sites of PHC contracting.

The selection of cases followed the logic of replication, adopting common criteria related to social, political, and institutional conditions. The selection criteria for our case cities were: (i) broad coverage of primary healthcare services; (ii) large urban centers with high population density and the presence of social and territorial inequalities; (iii) state capacities both to provide health services directly and to implement structured contracting processes; (iv) prior identification of the contracting of private providers as part of the municipal strategy for expanding primary healthcare services; and (v) active presence of established evaluation systems and legal, managerial and social accountability mechanisms, including audit courts, public prosecutors, and participation councils.

São Paulo and Fortaleza are marked by large populations, respectively, 11.5 and 2.6 million inhabitants, pronounced social and territorial inequalities, extensive PHC networks and consolidated contractual governance structures, making them suitable settings for analyzing how equity can be integrated into evaluation systems in complex health systems.

Both cities operate within the SUS and rely extensively on contractual arrangements with non-profit organizations for the management and delivery of publicly funded PHC services. Despite this common institutional framework, they differ in their contracting trajectories and governance models. Box 1 summarizes these features.

This comparative design allows examination of governance challenges across different contractual configurations within a shared institutional context, including a common constitutional and regulatory framework, the same national primary health care policy, similar contractual governance arrangements, and comparable accountability and participatory mechanisms.

### 2.3 Research process, data collection and analysis

The study was developed through four successive and interrelated stages, summarized in Figure 1. The sources associated with each stage (Figure 1) were used sequentially and iteratively to connect the analysis of contractual arrangements in São Paulo and Fortaleza with the development of a user-centered questionnaire that could be incorporated into the routine evaluation framework used in both cities. The sequential organization of the research stages served the iterative development of the analytical framework and questionnaire; it did not involve repeated measurement of the same participants, facilities or outcomes over time.

First, a mapping and policy analysis was undertaken in both municipalities. During this stage we examined: (a) the evolution of contractual and accountability arrangements; and (b) how coordination mechanisms and, especially, evaluation systems operate in practice. The analysis drew on legal and policy documents, municipal health plans, management contracts, evaluation manuals and institutional reports related to PHC governance in São Paulo and Fortaleza and was informed by categories and principles derived from the literature on contracting (2). Minutes of municipal and local health council meetings were systematically analyzed to identify recurring concerns, demands and experiences raised by users and councilors, particularly those related to quality of care, equity and the functioning of PHC services.

Also in this phase, semi-structured key informant interviews with public managers at the three levels (central, regional and unit), managers of different OSSs, health professionals of 16 PHC units (eight in each city) and health council representatives were held to map practices and perceptions around coordination mechanisms functioning and outputs. The PHC units visited were selected in collaboration with the respective Health Departments, based on two criteria: high social vulnerability and higher levels of social participation and activity of the councils. In total, 99 interviews were conducted: 39 in São Paulo in 2023; and 60 in Fortaleza between October 2023 and February 2024. Participants were selected through purposive sampling based on institutional roles and snowball sampling. For details on the interview guides and for participant profiles, see Supplementary material 1.

Interviews followed informed consent procedures, were audio-recorded and transcribed verbatim. Interviews addressed contracting arrangements, evaluation practices, informational tools and participation channels. Interview data were analyzed independently by at least two researchers. The data were organized into analytical summaries structured around vertical coordination (between bodies in central, regional and unit levels) and horizontal coordination (between bodies, managers, providers, professionals and councils at the same level) on three main governance domains: contracting, evaluation and participation. The analysis involved identifying recurrent themes through an iterative process combining deductive and inductive coding (2). The findings were subsequently discussed, refined, and validated in sessions with public officials, OSS managers and health councilors, as part of a collaborative process that led to the development of an Action Plan.

Second, as part of the Action Plan implementation we developed an analytical framework for equity in PHC - based on policy documents and the relevant literature - as well as a methodology to guide the focus groups. This framework, presented in Table 1 and developed as described in Section 3.2, was used as an analytical reference to assess the extent to which indicators included in Municipal Health Plans (MHPs) and OSS contracts captured equity dimensions. It also oriented the development of the questionnaire. The methodology used to design the focus groups drew on participatory approaches previously applied to the co-development of patient-reported measures and collaborative quality improvement in health care (e.g., (22–25)).

**Table 1:** A conceptual framework for equity in PHC.

Key dimensions for promoting equity in primary care	Subdimensions	Connection to equity
Responsiveness to population needs and territorial conditions	Guaranteeing access to services	By adapting the organization, schedules, and forms of access to the social conditions of the population, indirect exclusion is reduced and access proportional to needs is guaranteed.
	Participation and social control	Social participation promotes procedural justice and makes services more responsive to the needs of historically underrepresented groups.
	Community outreach and health promotion	Knowledge and action on social determinants promote collective health and responsive care.
Sociocultural dimensions of care	Sociocultural considerations	Respect for diversity and non-discrimination to ensure equal treatment within healthcare.
	Mitigating structural inequalities	Addressing the causes of structural disadvantage leading to unequal access and health, such as discrimination based on race, sexuality, disability, or homelessness, and directing services to historically marginalized groups.
	Welcoming and humanizing	Explicitly prioritizing time and resources for people in greater vulnerability puts the principle of equity into practice in daily care.
Comprehensive care for the person	Autonomy and empowerment of individuals	Sharing decision-making power reduces asymmetries between professionals and users and strengthens the ability of vulnerable groups to influence decisions that affect their health.
	Focus on family health	A longitudinal relationship with families allows for an understanding of contextual determinants and the adaptation of care to social realities.
	Prevention and promotion of patient health	By addressing social and environmental determinants before illness occurs, the unequal distribution of health risks and problems is reduced.
	Intersectoral care and coordination	Coordination between services reduces barriers to navigation within the system—which disproportionately affect the most vulnerable—and increases the problem-solving capacity of care.

Source: Prepared by the authors, NCSD, 2026.

Third, we then collected qualitative data through focus groups with service users and health council representatives. Focus groups were conducted to explore users’ experiences and identify dimensions of quality and equity not adequately captured by existing evaluation systems. In each municipality, from the initial eight PHC units, four were selected. A total of 15 focus groups were conducted with 82 participants, including 64 PHC users and 18 health council representatives. Participants were recruited through health councils, PHC units and local civil society organizations. For profile of participants of the focus groups, see Supplementary material 1, Table 2.

Discussions explored participants’ experiences of care within PHC services, inviting them to reflect on their care pathways before, during and after contact with services, identify situations of equity and inequity, and collectively prioritize dimensions for inclusion in PHC evaluation systems (For more details see Focus Group and Nominal Group Technique Guide, Supplementary material 1).

Focus groups followed informed consent procedures, were audio-recorded and transcribed verbatim. Thematic content analysis followed the stages of pre-analysis, exploration and interpretation (26), combining deductive categories derived from the equity framework (Table 1) with inductive refinement based on participants’ discussions.

Building on the documentary analysis, council meeting minutes, interviews and focus group findings, the research team linked the issues prioritized by users and health council representatives to the equity-related dimensions identified in Table 1. This matrix informed an initial questionnaire, designed to capture dimensions of quality, access, communication, continuity, respect and equity that were insufficiently represented in existing evaluation systems. The process followed general principles for the development of valid and feasible indicator sets, balancing conceptual relevance with practical applicability in routine evaluation systems (27).

The draft instrument was refined through collaborative workshops with public managers and OSS managers, focusing on the clarity, relevance, feasibility and potential integration of the questions into routine PHC evaluation processes.

Fourth, questionnaire testing and refinement are currently underway through a collaborative process involving researchers, PHC managers and implementation partners. This phase includes planned semantic validation to assess respondents’ understanding of the questionnaire items, followed by psychometric testing to evaluate the instrument’s measurement properties. During this phase we are also exploring opportunities to embed the questionnaire within existing PHC evaluation routines.

## 3 Results

This section presents the findings from the different stages of the study and the ways in which they informed one another throughout the co-creation process.

### 3.1 Existing evaluation systems: what is measured and what remains invisible?

The co-creation process developed in this study emerged within health systems that already possessed relatively sophisticated arrangements for monitoring, evaluating and governing primary health care services. Analysis in São Paulo and Fortaleza revealed sustained efforts to strengthen monitoring and performance assessment, alongside persistent challenges in capturing dimensions of care quality, user experience and equity.

#### 3.1.1 Evolution of evaluation systems in contracted PHC

Over the past two decades, both São Paulo and Fortaleza have expanded the participation of non-state actors in the provision of primary health care services while also adapting the mechanisms used to monitor, evaluate and govern service delivery. These trajectories were not linear. They involved political contestation, administrative constraints, external oversight pressures and successive attempts to reconcile service expansion with public accountability requirements.

In São Paulo, partnerships with OSSs became the predominant arrangement for PHC provision. As contractual governance expanded, the municipality developed more formalized mechanisms for monitoring contracts, defining targets and assessing provider performance. The territorial organization of contracts, meaning that one OSS is responsible for managing and delivering services offered by public primary care units in each health region, facilitated comparisons across providers operating in different parts of the city and contributed to the consolidation of a structured performance management framework (28). For several years, however, the core architecture of the municipal evaluation system remained relatively stable and centered on production, operational and compliance-oriented indicators. More substantial revisions emerged only after growing scrutiny from oversight institutions and broader debates regarding the adequacy of existing metrics for assessing service quality and health outcomes. Recent reforms therefore sought to broaden evaluation beyond service production and operational performance, incorporating selected quality-related and monitoring indicators, although challenges remain regarding data integration, territorial adaptation and the effective use of performance information for decision-making.

Fortaleza followed a different trajectory. The municipality did not adopt a territorially dominant model of contracted PHC provision comparable to São Paulo. Instead, it developed a more segmented arrangement in which direct public provision and OSS management coexist in relation to core PHC service delivery, while care-related macroprocesses were contracted separately and transversally across the municipal network. This macroprocess contract includes functions such as reception, scheduling, pharmacy logistics, laboratory flows, medical records and information systems. In a context of political resistance to outsourcing core PHC activities, this arrangement allowed the municipality to contract managerial and support functions across the network while expanding the contracting of clinical service delivery more gradually and selectively. It also contributed to the universalization of electronic health records and the consolidation of business intelligence tools, creating a more integrated information infrastructure for monitoring and coordination.

Despite these differences, both municipalities exhibit an expansion of evaluation capacities associated with contracted PHC governance. Evaluation systems became increasingly oriented toward tracking service production, coverage, operational performance, contractual compliance and selected clinical indicators. By contrast, dimensions related to quality of care, user experience, responsiveness and equity have generally received considerably less attention within municipal routine evaluation frameworks. While these dimensions are not entirely absent from existing systems and have gained visibility in recent reform efforts, they remain comparatively underdeveloped, inconsistently measured and insufficiently incorporated into performance assessment processes.

#### 3.1.2 Accountability arrangements in contracted PHC

Across both municipalities, the governance of contracted PHC combined mechanisms associated with managerial, legal and social accountability.

Managerial accountability occupied a central position within both systems through contractual indicators, targets, reporting requirements and performance monitoring practices. In São Paulo and Fortaleza, provider performance was routinely assessed through formal contractual mechanisms that generated a continuous flow of data on coverage, operational performance, and compliance with agreed commitments.

Legal accountability was exercised through courts of accounts, public prosecutors and other oversight institutions responsible for scrutinizing contractual arrangements and the use of public resources. These actors influenced the evolution of monitoring and evaluation systems through audits, reviews and recommendations concerning contractual governance, performance indicators and accountability procedures. In São Paulo, external scrutiny contributed to revisions of the evaluation framework and stimulated debates regarding the adequacy of existing indicators for assessing service quality and performance.

Social accountability was expressed through participatory institutions such as health councils, health conferences, ombudsman services and other mechanisms for community engagement. These spaces provided opportunities for users and community representatives to discuss service provision, raise concerns and influence health policy decisions. Nevertheless, interviews and document analysis pointed to persistent challenges affecting these arrangements, including uneven participation, difficulties in renewing and broadening representation, and perceptions that citizen contributions had limited influence on managerial and contractual decisions. While these mechanisms generated valuable information regarding users’ experiences and service needs, such information was rarely translated into systematic inputs for provider monitoring, performance assessment or contractual evaluation.

More broadly, the analysis suggests that social accountability mechanisms remained only partially connected to the formal systems used to monitor and evaluate provider performance. While citizen participation was present within the broader governance architecture of PHC, information generated through participatory channels was not systematically incorporated into contractual monitoring frameworks or routine performance assessment processes. This separation between participatory and evaluative arrangements emerged as one of the key institutional challenges identified by the first phase of the study and informed the development of the Action Plan. The Plan, discussed in both municipalities with public officials, professionals and health councilors, established a shared objective that contractual performance metrics should capture not only service delivery outputs, but also the capacity of health teams to understand and respond to local realities, thereby supporting more equitable care.

### 3.2 Citizen perspectives: what dimensions of care matter but remain unmeasured?

The implementation of the Action Plan started with the development of the equity-focused conceptual framework. Rather than adopting a pre-existing taxonomy, we constructed the framework by articulating Brazilian policy frameworks - which define equitable care as recognizing and responding to differences in life and health conditions (29)^1^ - with the international literature on health equity (10, 30, 31).

We mapped areas of PHC in which socially produced and potentially unjust differences could shape users’ access to, experience of, or ability to benefit from care, and in which health services could plausibly intervene to prevent or mitigate such differences. Relevant elements were grouped and systematized into three broad analytical dimensions: responsiveness to population needs and territorial conditions, sociocultural dimensions of care, and comprehensive care for the person as can be seen in Table 1.

The analysis of the equity dimensions in MHPs and OSS contracts showed a marked difference in evaluative emphasis: while indicators in municipal health plans addressed these dimensions relatively broadly, contractual indicators showed substantially more limited coverage of them. For example, in São Paulo, 50 of the 57 indicators in the MHP are directly or indirectly linked to the “comprehensive care for the person” dimension of equity; however, in the contracts governing service provision, only one of 15 indicators bears any relation to this dimension.

In the third stage, the focus group discussions explored how the analytical dimensions presented in Table 1 are experienced and prioritized by users and health councilors. Across both municipalities, participants described barriers and everyday interactions with PHC services, providing concrete illustrations of how quality, access, communication, continuity, respect and equity are enacted in practice. These experiences helped confirm that these dimensions remain underrepresented in existing evaluation systems.

#### 3.2.1 Responsiveness to population needs and territorial conditions

Across both municipalities, discussions on access centered on waiting times, appointment scheduling, referrals, exams, medicines, and system navigation. Participants described long waits for assessments and specialist care “I’ve been on the waiting list for an assessment for my son for almost two years” (service-user FG 3) and “I’ve been on the waiting list to see a vascular specialist for almost a year” (service-user FG 7) - as well as difficulties in referral pathways — “If it’s far away, I won’t go… we have specialists nearby” (service user, FG 4) — and uncertainty about priority criteria — “Oh, they’ll only see people who are really sick.” (service-user, FG 1). Access was thus experienced not simply as service availability, but as the ability to obtain timely care and navigate care pathways.

Communication, information, and transparency were also central. Participants reported difficulties obtaining information about waiting lists, referrals, service flows, and administrative changes. Digital tools were sometimes seen as helpful but also as exclusionary due to limited access, low digital literacy, and system instability. As one participant noted “I don’t even have a computer, I don’t know how to use one… Even if I manage to get online, I won’t understand what is written” (service-user, FG 8).

Territorial conditions further shaped access. Participants highlighted insecurity around health facilities and queues, shortages of medicines and supplies, and the impact of mobility constraints and socioeconomic vulnerability. As described by one participant “The danger is greater for those who arrive at dawn to join the queue. That’s when muggers pass by and take everyone’s phones” (service-user, FG 2). Participants also emphasized the need for adapted access for people with disabilities, including transport support and home visits. These accounts underline how territorial vulnerability shapes service use and equity.

Finally, participants reported limited access to decision-makers and weak participatory channels. “It’s very difficult to get to a supervisor to have a conversation…” (service-user FG 12) was a common concern, alongside limited feedback from councils and low awareness of participatory structures. At the same time, some participants described positive experiences of collective mobilization leading to local improvements, suggesting that participatory mechanisms are valued but unevenly effective and accessible.

Overall, the Responsiveness to Population Needs and Territorial Conditions dimension is grounded in concerns about waiting times, scheduling, referral pathways, information, digital access, material conditions, safety, and participation.

#### 3.2.2 Sociocultural dimensions of care

Beyond access and territorial conditions, discussions on communication, respect, and interpersonal aspects of care highlighted the relational dimensions of care and how services were experienced during interactions with health professionals.

When reflecting on their interactions with health professionals, participants emphasized the importance of feeling heard and respected, and many reported positive experiences with primary care physicians in PHC services. As stated by one focus group participant: “The doctor is a blessing” (service-user, FG 4).

At the same time, some of the most significant interpersonal challenges were not necessarily located in the clinical encounter itself, but in organizational routines and administrative flows, as illustrated by the following account: “Sometimes they already know there are no more slots in a team, they should go outside and inform us… but no, people stay in the queue, and only when they get inside the health unit they’re told it’s over… Next day, you must start all over again from scratch” (service-user, FG 3).

Consequently, even when the doctor–patient relationship is perceived as strong, failures in organizational routines and in communication about service processes can undermine users’ confidence and willingness to seek care in the future. In this sense, experiences of respectful treatment were frequently described as a key component of care quality. As noted by one participant: “I felt welcomed… she went to check how many people were ahead of me in the queue” (service-user, FG 6).

Conversely, communication failures, lack of attention, or dismissive interactions could negatively affect users’ trust in services and their willingness to seek care, as illustrated by another participant: “I cry and leave” (service-user, FG 1).

Participants also highlighted the importance of recognizing differences among users and adapting care accordingly. They referred to situations involving older adults, people with disabilities, and individuals facing distinct social and economic circumstances, suggesting that care quality depends not only on the services offered but also on the ability of professionals and organizations to communicate effectively and respond appropriately to diverse needs. As one participant noted: “Sometimes he says things that I don’t quite understand” (service-user; FG 12).

These concerns also extended to the ability of services to establish trust and maintain meaningful relationships with users over time. Taken together, these findings indicate that service-users’ experiences of equity and quality were strongly shaped by interpersonal dimensions of care, including communication, listening, respect, and responsiveness to individual circumstances.

#### 3.2.3 Comprehensive care for the person

When invited to reflect on comprehensive care, continuity, coordination, and user-centered care, participants frequently referred to aspects of care that extended beyond individual consultations or isolated service encounters. Discussions highlighted the importance of services being able to accompany service users over time, maintain continuity in care processes, and respond to health needs in a coordinated manner. Experiences of fragmented care, repeated referrals, interrupted follow-up, and difficulties obtaining information about subsequent stages of treatment were often cited as barriers to effective care.

Across discussions on continuity of care, participants frequently emphasized the value of being followed by professionals who were familiar with their history, circumstances, and previous care needs. Conversely, focus group accounts highlighted frequent professional turnover, difficulties in scheduling follow-up appointments, and disruptions in care trajectories, expressed in statements such as “the daughter becomes the medical record” (service-user FG 5) and “even family doctors don’t stay in the periphery” (service-user, FG 6). Participants also reported situations in which users themselves assumed responsibility for navigating complex care pathways and connecting information across services.

Discussions further highlighted the importance of understanding service users as individuals with interconnected health and social needs. References to family circumstances, living conditions, and broader life contexts frequently emerged when participants reflected on what constitutes good-quality care. These accounts suggest that service users valued approaches capable of recognizing the complexity of their needs and integrating different aspects of care into coherent and meaningful care pathways.

Taken together, these findings indicate that service users associated quality care with continuity, coordination, and the ability of services to understand and respond to the whole person.

### 3.3 From user perspectives to evaluative dimensions

The focus groups were designed both to collect service-users’ perspectives on PHC services and as part of a co-creation process aimed at informing the development of evaluative dimensions and indicators to complement existing monitoring frameworks. This objective was particularly relevant because many of the dimensions identified by participants as central to their care experiences remain weakly represented in current evaluation systems.

The analytical framework (Table 1) provided a structure for organizing and interpreting the material produced in the discussions. Rather than introducing new categories, the focus groups made it possible to specify how evaluative concepts such as quality, equity, and responsiveness are experienced in practice and to identify the concrete situations through which they become visible to service-users.

The discussions further enabled participants to go beyond reporting experiences and to reflect on what should be observed and assessed. Rather than positioning citizens solely as respondents to researcher or manager-defined questions, the process engaged them in reflecting on what should be measured, why it matters, and how key dimensions of care should be understood and interpreted.

The co-creation process also highlighted differences between managerial and experiential knowledge. While managers and providers hold structured information on service organization and performance, service-users bring perspectives on barriers to access, communication, and care navigation that are often less visible in formal systems. The process therefore supported the articulation of citizen perspectives into evaluative dimensions capable of informing monitoring and decision-making.

Equity emerged as a cross-cutting principle rather than a separate domain, shaping how multiple dimensions of care were interpreted.

#### 3.3.1 From co-created dimensions to a proposed questionnaire

The co-creation process generated a set of proposed dimensions to guide the development of a citizen-informed questionnaire. These dimensions were translated into a preliminary integrated questionnaire designed to capture equity-related aspects of PHC performance that may complement existing monitoring frameworks by incorporating users’ perspectives.

Focus group findings were transformed into questionnaire items (Table 2) through an iterative process of qualitative analysis, item development, and successive rounds of refinement. This process operationalized key evaluative concepts - including equity, responsiveness, communication, and continuity of care - into measurable constructs suitable for routine assessment, while ensuring conceptual coherence and applicability across diverse contexts.

**Table 2:** Proposed dimensions for integrating user perspectives into PHC evaluation.

Key dimensions for promoting equity in primary care	Sub-dimensions	Items
Responsiveness to population needs and territorial conditions	Guaranteeing access to services	I need to arrive at the unit before opening time to be able to be seen on the day.
		My access to the health unit has already been hampered by feelings of insecurity.
		The legislation provides for priority service for people with disabilities, the older adults, pregnant women, breastfeeding mothers, people with infants, obese people, and people with reduced mobility. In this unit, these priorities are respected.
	Participation and social control	When I file a complaint with the ombudsman, I receive a response regarding the problem.
		The health council helps to improve the functioning of this health unit.
		I would like to contribute to and participate in decisions regarding my health unit.
	Community outreach and health promotion	It is easy to participate in the groups, for example, conversation or physical activity groups, organized by this unit.
Socio-cultural dimensions of care	Sociocultural considerations	I have already been discriminated against when receiving care at a health unit.
	Mitigating structural inequalities	I can get an appointment at the health unit after 7:00 p.m. and on weekends.
		The professionals at the unit understand the difficulties I face daily in taking care of myself.
	Welcoming and humanizing	I feel welcomed by the professionals at the health unit.
		The health unit is organized and well-maintained.
		Using the scheduling app, either by phone or computer, has made it easier for me to access the health unit.
		The health unit is prepared to receive people with mobility difficulties.
Comprehensive care for the person	Autonomy and Empowerment of Individuals (Interest in the Patient)	During consultations, the professionals listen attentively to what I say.
		During appointments, I understand what the professionals explain about what I should do to take care of my health.
	Focus on Family Health	The health unit contacts me when they need to speak with me or my family.
	Prevention and Promotion of Patient Health	I receive support from the Community Health Agent (ACS) to take care of my health.
	Intersectoral care and coordination	At this health unit, professionals help you access other public services in addition to healthcare.

The questionnaire items were formulated as concise statements describing concrete aspects of users’ experiences with PHC and were designed for response on a five-point Likert agreement scale, ranging from strongly disagree to strongly agree. Item development sought to minimize common response biases by prioritizing simple and accessible language, avoiding compound statements and, where possible, anchoring questions in observable situations and experiences. To help identify and limit acquiescence bias, three items were intentionally negatively worded and will therefore be reverse-scored in the analysis: “I need to arrive at the unit before opening time to be able to be seen on the day”; “My access to the health unit has already been hampered by feelings of insecurity”; and “I have already been discriminated against when receiving care at a health unit.”

The co-creation process thus produced a structured approach for translating service-user perspectives into evaluative dimensions and indicators. This process enabled different actors to contribute to the identification and operationalization of what should be evaluated and how it should be measured, while also fostering a shared basis for discussing quality and equity across institutional perspectives.

## 4 Discussion

The findings presented in this study contribute to ongoing debates regarding governance, accountability, and evaluation in plural health systems by showing that the development of increasingly sophisticated monitoring and assessment systems does not necessarily translate into a greater capacity to capture dimensions related to quality of care, user experience, and equity (2).

Such limitations reflect broader institutional dynamics regarding how evaluation systems are structured, which forms of knowledge become visible within routine assessment processes, and whose perspectives are considered legitimate for informing decision-making. In contexts where monitoring systems are strongly shaped by contractual governance and accountability requirements, dimensions that are easier to standardize and compare tend to receive greater attention than aspects of care that are more contextual, relational, and difficult to quantify (11).

Against this background, the co-creation process described in this paper offers insights into how user perspectives may contribute not only to strengthening participation, but also to broadening the range of dimensions incorporated into health system evaluation (32). By engaging users in reflecting on what should be observed and assessed, the process created opportunities to translate experiential knowledge into forms that can complement existing monitoring arrangements and make otherwise less visible aspects of care more actionable within institutional settings.

### 4.1 Evaluation, contracting and the transformation of state capacities

In both São Paulo and Fortaleza, contracting generated demands for monitoring, regulation, information management and performance assessment that required the development of new administrative and organizational capacities. These processes contributed to the reconfiguration of state capacities in response to new governance arrangements.

The trajectories observed in the two municipalities illustrate different patterns of this transformation. In São Paulo, the expansion of OSS management was accompanied by the consolidation of contractual governance structures and increasingly formalized mechanisms for performance monitoring and evaluation. Over time, the municipality developed significant capacities in contract management, target setting, performance assessment and regulatory oversight. At the same time, the predominance of contracted provision reduced the role of direct public management in the operation of PHC services.

Fortaleza followed a different path. Direct public provision remained the dominant model for PHC service delivery, while contractual arrangements expanded more selectively in the provision of primary care services, with greater emphasis on peripheral territories. At the same time, contracting was adopted across the PHC network for system-wide macroprocesses. This configuration allowed capacities developed within contractual governance - particularly information systems, monitoring routines and performance management tools - to coexist with and influence directly managed services.

These transformations also shaped the types of information produced by the system and the forms of performance prioritized for management and accountability. Across both municipalities, contractual governance was associated with indicators, targets and reporting routines centered on service production, coverage, operational performance and compliance. While these indicators respond to legitimate demands for transparency and oversight in contexts involving public-private arrangements, they also tend to privilege dimensions that are easier to measure and compare. By contrast, dimensions such as quality of care, user experience, responsiveness and equity depend on contextual and relational factors that are more difficult to capture through administrative data and contractual reporting. As a result, evaluation systems may become increasingly sophisticated in some domains while remaining limited in others (11).

Interviews suggest that these evaluative arrangements also influence how services are organized in practice. Activities that are clearly specified, routinely monitored, and linked to performance targets and payment models tend to attract greater managerial attention, whereas practices that are less readily measurable risk becoming less visible within organizational priorities. Participants associated this dynamic with an emphasis on production targets and standardized procedures, alongside concerns about reduced space for community engagement, health promotion and other activities that are harder to quantify.

These findings do not imply that contracting or performance management necessarily undermine such practices. Rather, they suggest that evaluation systems generate incentives that shape organizational attention and resource allocation. The challenge is therefore not only to improve measurement, but to ensure that what is measured reflects the broader objectives of primary health care. In this sense, expanding evaluation frameworks to include dimensions of quality, equity, responsiveness and citizen experience may help rebalance these incentives.

From this perspective, the development of equity-sensitive and user-centered indicators can be understood as part of a broader process of institutional learning (33). Such measures aim to address informational gaps created by the historical focus on dimensions that are easier to quantify, compare and audit. Strengthening evaluative frameworks in this direction can therefore be seen as part of reinforcing state capacity under increasingly complex governance arrangements.

### 4.2 Accountability imbalances and the limits of existing evaluation systems

As seen earlier, evaluation frameworks are shaped by the interaction of different accountability logics, each of which privileges particular forms of information, evidence, and performance assessment (34). The patterns observed in São Paulo and Fortaleza suggest that these logics are not symmetrical: while some accountability domains reinforce one another and become embedded in routine evaluation processes, others remain only partially integrated into systems of performance monitoring and decision-making.

Evidence in both municipalities suggests that substantial institutional effort is devoted to data production, reporting, and auditing activities. By contrast, fewer resources appear to be dedicated to the interpretation of information and its translation into planning and organizational learning. The Fortaleza case is particularly illustrative, where contractual governance structures integrated extensive auditing and verification functions into routine management. The findings from São Paulo indicate that this trajectory is not fixed. Although early evaluation frameworks were strongly centered on production and compliance indicators, subsequent reforms were driven in part by pressure from oversight institutions, which argued that existing metrics were insufficient to assess broader dimensions of performance, especially concerning quality. In this sense, legal accountability can function not only as a force of consolidation, but also as a driver of reform.

In contrast, social accountability remains insufficiently integrated into formal evaluation systems. Health councils, ombudsman services, and other participatory mechanisms generate relevant information on users’ experiences and needs, but this information rarely circulates into planning bodies, contractual monitoring frameworks or performance assessment routines. The main challenge, therefore, is not the absence of participatory inputs, but the lack of institutional mechanisms to translate them into evaluative information and incorporate that information in decision-making processes.

This imbalance suggests that the central issue is not a deficit of accountability, but differences in the capacity of managerial, legal and social accountability mechanisms to shape evaluation processes and the medium- and long-term development of the health system. While managerial and legal accountability tend to converge through shared indicators and reporting routines, social accountability operates through distinct forms of knowledge and communication. Its integration into evaluation systems therefore requires deliberate institutional mechanisms capable of translating citizen perspectives into information that is both meaningful and operationally usable (12, 13, 15).

From this perspective, the co-creation process described in this study can be understood as an attempt to bridge this gap. Rather than replacing existing accountability arrangements, it seeks to strengthen their articulation by creating channels through which user-generated information can be incorporated into routine evaluation and decision-making processes.

### 4.3 Co-creation towards more responsive evaluation systems

The findings suggest that the contribution of participatory approaches to health system governance extends beyond their conventional roles in representation, consultation, or social control. The co-creation process analyzed in this study indicates an additional function: the production of arrangements that shape how evaluative knowledge itself is generated. From this perspective, participants were not only asked to report experiences, but also to deliberate on what should be observed and assessed. In doing so, they contributed to defining the boundaries of what counts as relevant knowledge for evaluation and to capturing concrete equity dimensions (35).

Social accountability mechanisms often produce information that remains fragmented and weakly connected to managerial indicators and performance frameworks. The results therefore highlight not only a gap between participation and evaluation, but also the absence of structured mechanisms through which experiential knowledge can be systematically incorporated into evaluation processes.

This gap reflects the coexistence of distinct but complementary forms of knowledge. While managers and providers rely on administrative, operational, and performance data, users engage directly with dimensions of care that are less visible in formal systems, such as navigation barriers, communication processes, trust, continuity, and responsiveness. The challenge is not to privilege one form of knowledge over another, but to establish mechanisms through which they can be brought into dialog to capture and assess the complexity of health system performance.

This process suggests that co-creation can work as an institutional arrangement that connects experiential and managerial knowledge, enabling shared evaluative frameworks across actors occupying different institutional positions and perspectives (33).

The contribution of co-creation therefore lies in its capacity to make participation constitutive of evaluation, rather than merely supportive of it. When citizen perspectives are systematically incorporated into evaluative frameworks, they can strengthen the links between service experience and managerial action. In this sense, more responsive evaluation systems may support a more integrated cycle in which participation informs evaluation, evaluation informs decision-making, and improvements in services reinforce the value of participation.

From this point of view, user-centered and equity-sensitive indicators should be understood as complementary components of evaluation systems that expand what can be observed and acted upon. More responsive evaluation systems are therefore not those that reduce measurement, but those that broaden the range of relevant dimensions considered in assessing performance. This is particularly relevant in contexts marked by territorial and social inequalities, where aggregate indicators may obscure important variation in how care is experienced.

### 4.4 Embedding indicators in the evaluation cycle

The focus groups helped operationalize concepts that matter to service users and translate them into equity-oriented evaluative dimensions. Although the discussion about the incorporation of this perspective into municipal evaluation systems remains at an early stage, we initially identified three promising entry points for embedding participatory assessment within existing PHC evaluation processes: integrating user-informed indicators into quality assurance and user experience instruments; incorporating them into routine performance reviews between health authorities and providers to support context-sensitive service improvement; and aligning them with emerging national initiatives to include user experience in PHC financing and evaluation frameworks.

Once validated and incorporated into routine evaluation, repeated application of the questionnaire could support both cross-sectional comparisons across providers, territories or population groups and longitudinal assessment of changes in users’ experiences over time.

Together, these pathways suggest that participatory evaluation can be incorporated into existing institutional arrangements to strengthen the responsiveness and equity orientation of PHC evaluation.

### 4.5 Implications, limitations and future directions

The findings of this study have implications that extend beyond the specific cases of São Paulo and Fortaleza. Although the evaluative framework was developed within the Brazilian Unified Health System (SUS), the challenges addressed - balancing performance management, accountability requirements, and responsiveness to users’ experiences - are common to many health systems characterized by governance arrangements involving public and non-state providers.

More generally, the study suggests that participatory approaches can contribute to health system evaluation not only by enhancing legitimacy or representation, but also by broadening the informational basis on which quality, equity, and performance are defined and assessed in complex plural health systems.

A key contribution of the study lies in the process through which it was developed. The study highlights the potential of structured and analytically oriented co-creation processes involving users, community representatives, healthcare professionals, managers, providers, and researchers to identify evaluative gaps and translate experiential knowledge into dimensions that can inform routine monitoring and assessment.

Within the SUS context, particularly in large urban municipalities where primary care is organized through complex combinations of direct provision and contractual arrangements, the approach developed here may offer a useful way of improving and integrating dimensions of quality, equity, and user experience into existing evaluation systems. Both São Paulo and Fortaleza illustrate how such systems operate under pressures related to territorial inequality, organizational complexity, and increasing demands for accountability.

Several limitations should be acknowledged. The study is based on two large urban settings and focuses on specific configurations of PHC governance and contracting that represent different models of PHC contracting. Participants and healthcare facilities were anonymized throughout the analysis. The findings do not allow for generalization across all health system contexts, nor do they establish causal relationships between governance arrangements and outcomes. In addition, the instrument remains under development: while co-creation and initial refinement have been completed, semantic and psychometric testing and full validation of the questionnaire are still ongoing. The findings therefore speak primarily to the feasibility and development of the approach, rather than the instrument itself, its potential effects or institutional performance. A further limitation relates to the institutionalization of participatory evaluation processes. Incorporating users’ perspectives into formal evaluation systems may introduce new tensions, including risks of bureaucratization, indicator proliferation, selective use of information, or the decoupling of participation from decision-making processes.

Future research should therefore examine not only the development and incorporation of user-centered indicators into routine evaluation systems, but also how such information is interpreted, acted upon and connected to managerial, legal and social accountability mechanisms. Comparative research across different health systems, provider arrangements and institutional settings is needed to assess which dimensions are context-specific and which may be transferable across contexts. Longitudinal studies could further examine whether repeated use of user-centered measures captures changes in service responsiveness and equity over time, while implementation research should investigate whether incorporating experiential information into performance review and contracting processes changes organizational behavior, managerial priorities or service delivery. These questions are particularly relevant to plural health systems seeking to combine public accountability with increasingly diverse arrangements for service provision.

## 5 Conclusion

This study explored how user perspectives can inform the evaluation of contracted primary health care services through a participatory co-creation process in two large Brazilian municipalities. Rather than proposing a new standalone model of assessment, the study demonstrated how existing evaluation systems can be complemented and improved by incorporating dimensions of care derived from users’ experiences.

The findings highlight persistent asymmetries in what is measured in routine evaluation systems, with a strong emphasis on production and performance indicators and limited attention to quality, equity and responsiveness. They also show that participatory processes can support the identification and operationalization of evaluative dimensions that are otherwise difficult to capture through administrative data alone.

Overall, the study suggests that integrating users’ perspectives into evaluation is less a matter of adding new instruments than of expanding what counts as relevant knowledge for assessing health system performance, thereby strengthening accountability in complex governance arrangements. Further research is needed to examine how these participatory approaches can be adapted across institutional contexts and whether their incorporation into routine evaluation and accountability processes translates into changes in organizational behavior, service responsiveness and equity.

## Data Availability

The datasets presented in this article are not readily available due to ethical and privacy considerations related to the protection of research participants. Requests to access anonymized data should be directed to the corresponding author and will be considered subject to applicable ethical and confidentiality requirements.

## Footnotes

1 Examples of laws, policies and programs include Law No. 8,142/1990 (36); the Family Health Strategy (Estratégia Saúde da Família) (29); the National Humanization Policy (Política Nacional de Humanização – HumanizaSUS) (37); the Strategic Affirmative Action Program: Black Population and AIDS (Programa Estratégico de Ações Afirmativas: População Negra e Aids) (38); and the Health Service Users’ Bill of Rights (Carta dos Direitos dos Usuários da Saúde) (39).

## References

[1] Núcleo de Cidadania, Saúde e Desenvolvimento (NCSD). SUS a plural Healthcare system. COMPLUS Series Brazil, vol. 1 São Paulo: CEBRAP (2025).

[2] Khan Z Haddad F Rao V Jith JR Breeze P Garimella S . Community participation and contracting between state and non-state actors in primary care: a scoping review of evidence. Int J Equity Health. (2025) 24:199. doi: 10.1186/s12939-025-02567-3 PMC12239432 40634992

[3] Liu X Hotchkiss DR Bose S. The effectiveness of contracting-out primary health care services in developing countries: a review of the evidence. Health Policy Plan. (2008) 23:1–13. doi: 10.1093/heapol/czm042, 17999986

[4] Loevinsohn B Harding A. Contracting for the Delivery of Community Health Services: A Review of Global Experience. Health, Nutrition and Population Discussion Paper. Washington, DC: World Bank (2004).

[5] Coelho VSP Szabzon F. "SUS: the Brazilian health care system". In: Powell M Agartan TI Béland D, editors. Research Handbook on Health Care Policy. Cheltenham, UK and Northampton, MA, USA: Edward Elgar Publishing (2024). p. 418–39.

[6] Ferreira-Batista NN Teixeira AD Diaz MDM Postali FAS Moreno-Serra R Love-Koh J. Is primary health care worth it in the long run? Evidence from Brazil. Health Econ. (2023) 32:1504–24. doi: 10.1002/hec.4676 37010114

[7] Massuda A Hone T Leles FAG De Castro MC Atun R. Brazilian health system at crossroads: progress, crisis and resilience. BMJ Glob Health. (2018) 3:e000829. doi: 10.1136/bmjgh-2018-000829 PMC6035510 29997906

[8] Coelho VSP Greve J. Social health organizations and the performance of the SUS: a study on basic healthcare in São Paulo. Dados. (2016) 59:867–901. doi: 10.1590/00115258201694

[9] Khan Z Haddad F Coelho VSP. "Universal access to health: is contracting services a solution?". In: Anais do 14º Encontro da Associação Brasileira de Ciência Política (ABCP). Salvador, Brazil: Associação Brasileira de Ciência Política (2024). Available online at: https://cienciapolitica.org.br/web/anais-do-14-encontro-da-abcp/

[10] Dover DC Belon AP. The health equity measurement framework: a comprehensive model to measure social inequities in health. Int J Equity Health. (2019) 18:36. doi: 10.1186/s12939-019-0935-0, 30782161 PMC6379929

[11] Lévesque J-F Haggerty J Beninguissé G Burge F Gass D Beaulieu M-D. Mapping the coverage of attributes in validated instruments that evaluate primary healthcare from the patient perspective. BMC Fam Pract. (2012) 13:20. doi: 10.1186/1471-2296-13-20, 22423617 PMC3353250

[12] Barboza PIB Vasconcellos M d P Aith FMA. Transparência nas relações público-privadas para prestação de serviços de saúde no estado de São Paulo. Cad Ibero Am Direito Sanit. (2021) 10:125–52. doi: 10.17566/ciads.v10i4.810

[13] Bispo JP Serapioni M. Community participation: lessons and challenges of the 30 years of health councils in Brazil. J Glob Health. (2021) 11:03061. doi: 10.7189/jogh.11.03061, 33828840 PMC8005309

[14] Coelho VSP. What did we learn about citizen involvement in the health policy process: lessons from Brazil. J Public Deliberation. (2013) 9:9. doi: 10.16997/jdd.161

[15] de Cássia Costa Da Silva R de Novais MAP Zucchi P. Social participation in the unified health system of Brazil: an exploratory study on the adequacy of health councils to resolution 453/2012. BMC Health Serv Res. (2021) 21:1120. doi: 10.1186/s12913-021-07161-1, 34666755 PMC8524792

[16] Chouinard JA. The case for participatory evaluation in an era of accountability. Am J Eval. (2013) 34:237–53. doi: 10.1177/1098214013478142

[17] Cargo M Mercer SL. The value and challenges of participatory research: strengthening its practice. Annu Rev Public Health. (2008) 29:325–50. doi: 10.1146/annurev.publhealth.29.091307.083824, 18173388

[18] Cornwall A Jewkes R. What is participatory research? Soc Sci Med. (1995) 41:1667–76. 10.1016/0277-9536(95)00127-s 8746866

[19] Bate P Robert G. Experience-based design: from redesigning the system around the patient to co-designing services with the patient. BMJ Qual Saf. (2006) 15:307–10. doi: 10.1136/qshc.2005.016527, 17074863 PMC2565809

[20] Greenhalgh T Hinton L Finlay T Macfarlane A Fahy N Clyde B. Frameworks for supporting patient and public involvement in research: systematic review and co-design pilot. Health Expect. (2019) 22:785–801. doi: 10.1111/hex.12888, 31012259 PMC6737756

[21] Correia D Mendes A. O Fundo de Saúde e sua apropriação pelas Organizações Sociais no município de São Paulo. Saude Debate. (2023) 47:76–89. doi: 10.1590/0103-1104202313705

[22] Blackburn S Higginbottom A Taylor R Bird J Østerås N Hagen KB. Patient-reported quality indicators for osteoarthritis: a patient and public generated self-report measure for primary care. Res Involv Engagem. (2016) 2:5. doi: 10.1186/s40900-016-0019-x, 29062506 PMC5611660

[23] Coteur K Matheï C Schoenmakers B Van Nuland M. Co-design to increase implementation of insomnia guidelines in primary care. Patient Educ Couns. (2022) 105:3558–65. doi: 10.1016/j.pec.2022.08.018, 36075810 PMC9425708

[24] Lin E Gobraeil J Johnston S Venables MJ Archibald D. Consensus-based development of an assessment tool: a methodology for patient engagement in primary care and CPD research. J Contin Educ Heal Prof. (2022) 42:153–8. doi: 10.1097/CEH.0000000000000440, 35916890 PMC9398503

[25] van der Merwe M D’Ambruoso L Witter S Twine R Mabetha D Hove J . Collective reflections on the first cycle of a collaborative learning platform to strengthen rural primary healthcare in Mpumalanga, South Africa. Health Res Policy Syst. (2021) 19:66. doi: 10.1186/s12961-021-00716-y, 33874951 PMC8054125

[26] Bardin L. Análise de conteúdo. São Paulo: Edições 70 (2016).

[27] Schang L Blotenberg I Boywitt D. What makes a good quality indicator set? A systematic review of criteria. Int J Qual Health Care. (2021) 33:mzab107. doi: 10.1093/intqhc/mzab107, 34282841 PMC8325455

[28] Coelho VSP Marcondes LM Barbosa M. “Accountability” e redução das desigualdades em saúde: a experiência de São Paulo. Novos Estud CEBRAP. (2019) 38:323–49. doi: 10.25091/S01013300201900020003

[29] Ministério da Saúde. Portaria no 2.436, de 21 de setembro de 2017: Política Nacional de Atenção Básica [Ordinance No. 2,436 of 21 September 2017: National Primary Health Care Policy]. Brasília: Ministério da Saúde (2017).

[30] Carden F. Building evaluation capacity to address problems of equity. N Dir Eval. (2017) 154:115–25. doi: 10.1002/ev.20245

[31] Faruquzzaman O Tse S Cameron R Chee K Kirst M Wilson C. Approaches to incorporating equity into program evaluation: a scoping review. Eval Program Plann. (2025) 112:102648. doi: 10.1016/j.evalprogplan.2025.102648, 40652620

[32] Bledsoe KL Graham JA. The use of multiple evaluation approaches in program evaluation. Am J Eval. (2005) 26:302–19. doi: 10.1177/1098214005278749

[33] Valderas JM Porter I Martin-Delgado J Rijken M De Jong J Groene O . Development of the patient-reported Indicator surveys (PaRIS) conceptual framework to monitor and improve the performance of primary care for people living with chronic conditions. BMJ Qual Saf. (2025) 34:529–36. doi: 10.1136/bmjqs-2024-017301, 39174334

[34] Coelho VSP. "Governança em Saúde: em busca de padrões". In: Fiore M Dolhnikoff M, editors. Mosaico de Olhares. São Paulo: SESC (2021). p. 93–112.

[35] Segone M. Evaluation for equitable development results. New York: UNICEF Evaluation Office (2012).

[36] Brazil. Lei n° 8.142, de 28 de dezembro de 1990 [Law No. 8,142 of 28 December 1990]. Dispõe sobre a participação da comunidade na gestão do Sistema Único de Saúde (SUS) e sobre as transferências intergovernamentais de recursos financeiros na área da saúde e dá outras providências. Brasília: Presidência da República (1990).

[37] Ministério da Saúde. HumanizaSUS: Documento base para Gestores e Trabalhadores do SUS [HumanizaSUS: National Humanization Policy—Reference Document for SUS Managers and Workers]. 4th ed. Brasília: Editora do Ministério da Saúde (2010).

[38] Ministério da Saúde, Secretaria de Vigilância em Saúde, Programa Nacional de DST e Aids. Programa Estratégico de Ações Afirmativas: População Negra e Aids [Strategic Affirmative Action Programme: Black Population and AIDS]. Brasília: Ministério da Saúde (2005).

[39] Ministério da Saúde. Portaria n° 1.820, de 13 de agosto de 2009: Carta dos Direitos dos Usuários da Saúde [Ordinance No. 1,820 of 13 August 2009: Health Service Users’ Bill of Rights]. Brasília: Ministério da Saúde (2009).

