# Supplementary material for: Accountability and equity in primary health care: co-creating user-centered evaluation systems for public-private partnerships
Source: Front Public Health. 2026 Sep 29;14:1938730. doi: 10.3389/fpubh.2026.1938730 (PMC13658590; doi:10.3389/fpubh.2026.1938730)
Supplement: Supplementary file 1 [file Data_Sheet_1.PDF]

CEBRAP
centro brasileiro de análise e planejamento

Annex 1 –

1. Semi-structured interviews Guides

1. 1 Interview Guide for the Municipal Health Department (MHD) and Social Health Organizations (SHO)

Brazilian Center for Analysis and Planning – CEBRAP
Principal Investigator: Dr. Vera Schattan Pereira Coelho

Questionnaire No. ______________________ Interview Date:__________________
Interviewer's name:____
Start time: ___________________End time: __________________

Interviewee identification
Identification:____
Category: ____

Interviewee profile
a) Age
b) Education
c) Current position
d) Career path leading to their current position

Questions

1. **[For MHD]** Could you describe the implementation of Primary Health Care contracting and evaluation processes in the municipality?
2. **[For the SHO]** Could you share a bit of the history of this SHO? How was it organised? Who are its founders? What is its track record?
3. **[MHD and SHO]** Concerning the contracting of PHC services:
   a) What is your involvement in PHC evaluation processes?
   b) What metrics are used for the evaluation? Which ones are the most significant in your view?
   c) Have you participated in the definition of questionnaires and indicators? Could you briefly describe these experiences? Which sectors and entities were involved? What were the main challenges related to defining these questionnaires and indicators?
   d) How do you see the relationship between evaluation and the type and quality of services provided?
   e) Did changes occur in the provision of services?
   f) What difficulties do you recognise in the contracting process?
   g) What needs to be improved in the evaluation processes?
   h) What contributions can [SHO/MHD] make in this regard?
4. **[MHD and SHO]** Is the implementation of the contracting model proceeding uniformly across all regions of the city? Is there variation between regions? If

there are variations, what difference does the SHO make? And does user involvement make a difference?

5. **[MHD and SHO]** Regarding the implementation of instruments to capture users' perceptions, how is this progressing in the municipality? Is implementation occurring uniformly across all regions of the municipality? What difficulties and challenges are related to the implementation of these instruments?
6. **[MHD and SHO]** The contract evaluation process can help foster innovation in service delivery. How?
7. **[MHD and SHO]** In addition to internal evaluations and oversight bodies—such as the Ombudsman's Office and the Court of Auditors—is there any evaluation process involving users? And do Health Councils conduct any type of monitoring?
8. **[MHD and SHO]** Are patients and advisors invited to participate in discussions regarding the quality and suitability of the services provided? If so, is this information taken into account during the processes of evaluating, contracting, and renewing contracts with providers? If not, do you see opportunities to advance practices in this area?
9. **[MHD and SHO]** How is the analysis and/or use of the collected data carried out? What are the main difficulties?
10. **[For the SHO]** Does the SHO make data regarding its evaluation available through any public channel?

1.2 Interview Guide for Citizen Representatives (Health Council members)

Brazilian Center for Analysis and Planning – CEBRAP
Principal Investigator: Dr. Vera Schattan Pereira Coelho

Questionnaire No. ______________________ Interview Date:__________________
Interviewer's name:____
Start time: ___________________End time: __________________

Interviewee identification
Identification:____
Category: ____

Interviewee profile
a) Age
b) Education
c) Current position
d) Career path leading to their current position

Questions

1. There has been an increase in the use of management contracts in services offered

by Primary Health Care (PHC) in the municipality. How do you evaluate this process?

2. Was this expansion uniform across all regions?
    a) Social mobilization present in the region affect the way management contracts are implemented?
    b) Do you recognize differences in implementation related the SHO profile?
3. Do you believe that the councils can be useful in improving the contracting process?
4. From your perspective, can health councils foster innovation in the contract evaluation process?
5. Are patients and councillors invited to participate in discussions regarding the quality and suitability of the services provided? If so, is this information taken into account during the processes of evaluating, contracting, and renewing contracts with providers? If not, do you see opportunities to advance practices in this area?

1.3 Focus Group and Nominal Group Technique Guide

A) Welcome, Introduction of the Research Team, and Participant Introductions
A signature on the informed consent form was requested. The research team was introduced first, followed by introductions from the participants, who were invited to state their name and share what they most liked to do in their free time. This activity was intended to reduce tension and facilitate interaction, particularly if participants were not previously acquainted with one another.

B) Study Presentation
The research team provided a brief presentation of the study, including its objectives, the purpose of the activity, and the importance of user participation in identifying aspects relevant to the evaluation of Primary Health Care.

C) Presentation on Health Indicators and Health Care Evaluation
A brief presentation was given on what health indicators are and how they are used, addressing their concept, purpose, and application in monitoring and evaluating health services. Examples of how health care evaluation is currently conducted by the municipality were then presented to provide context for the discussion and support participants' contributions.

D) Focus Group Discussion (30 minutes)
Following the initial contextualisation, a focus group discussion was conducted based on guiding questions addressing participants' experiences with the health service, including: a marked experience (positive or negative) in their interaction with the health facility; how their perception of their own health had changed since they began receiving care at this facility, and what had contributed to this. The discussion was structured around the user's journey through the health service, from the period preceding service utilisation to the period following the consultation or service encounter. The purpose of these questions was to identify, from the participants' perspectives, aspects considered relevant for inclusion in the evaluation of PHC.

Throughout the discussion, participants were encouraged to freely share their experiences, perceptions, and suggestions.

E) Nominal Group Technique (30 minutes)

Following the focus group discussion, a Nominal Group Technique (NGT) was applied to prioritise evaluation topics. Each participant was individually invited to suggest one or two topics they considered should be included in the evaluation of PHC contracts but were not currently measured. Suggestions were then shared with the group and grouped on a shared panel. Participants subsequently prioritised the topics through an individual voting process, selecting those they considered most relevant to assessing equity in Primary Health Care. The voting results were used to identify priority topics from the perspective of users and user representatives on health councils, contributing to the definition of the dimensions and aspects considered in the development of the questionnaire.

F) Closing

At the end of the activity, the research team provided a summary of the main topics discussed and asked participants whether they agreed with the summary or wished to add anything further. The team reaffirmed the confidential and aggregated use of the information shared, thanked participants for their contributions, and provided information about the next steps of the research.

2. Tables - 2.1Table 1, Phase 1; Professional role and level of the interviewees

Level	Function	Gender		Total = 99
		Male	Female	
Municipal Health Department (central level)	Management, Epidemiology and Information	4	9	13
Regional Health Coordination Office	Management, contracts management, Primary Health Care, Epidemiology and Information, Capacity building, Human resources, Health programs	3	12	15
Technical Health Supervision	Management contracts, management, social participation, development, Epidemiology and Information, Primary Health Care, Capacity building,	10	22	32
Primary Health Care Unit	Management, Health professional	6	10	16
Health Councils Central, regional and local	Health councillor	7	10	17
Social Health Organization	Management	1	5	6

Source: NCSD (2026).

2.2 Table 2, Phase 2; Focus Groups, participants profile

Variables	Councillors, user representative (n=18)		Users (n=64)	
	N	%	N	%
**Sex**				
Female	12	14.63	51	62.19
Male	6	7.31	13	15.85
**Age**				
Young person (18 to 29 years old)	0	0.0	9	10.97
Adult (30 to 59 years old)	8	9.75	25	30.48
Elderly (60 years or older)	10	12.19	29	35.36
Not informed	0	0.0	1	1.21
**Race/Color**				
White	5	6.09	21	25.60
Brown	8	9.75	30	35.58
Black	4	4.87	12	14.63
Yellow	1	1.21	1	1.21
Not informed	0	0.0	1	1.21
**Education**				
Up to Incomplete High School Education	4	4.87	27	32.92
Completed High School	6	7.31	21	25.60
Higher education or postgraduate studies	7	8.53	10	12.19
**Work Situation**				
Retired / Pensioner	7	8.53	25	30.48
Active (Formal, Informal or Self-Employed)	7	8.53	18	21.95
Unemployed	3	3.65	19	23.17
Not informed	1	1.21	2	2.43

Source: NCSD (2026)
